# Influence of gadolinium, field-strength and sequence type on quantified perfusion values in phase-resolved functional lung MRI

**DOI:** 10.1371/journal.pone.0288744

**Published:** 2023-08-01

**Authors:** Julian Glandorf, Fynn Brunzema, Filip Klimeš, Lea Behrendt, Andreas Voskrebenzev, Marcel Gutberlet, Marius M. Wernz, Robert Grimm, Frank Wacker, Jens Vogel-Claussen

**Affiliations:** 1 Institute for Diagnostic and Interventional Radiology, Hannover Medical School, Hannover, Germany; 2 Biomedical Research in Endstage and Obstructive Lung Disease Hannover (BREATH), Member of the German Centre for Lung Research (DZL), Hannover, Germany; 3 MR Application Predevelopment, Siemens Healthcare GmbH, Erlangen, Germany; Erasmus MC Sophia Children’s Hospital, NETHERLANDS

## Abstract

**Purpose:**

The purpose of this study is to evaluate the influences of gadolinium-based contrast agents, field-strength and different sequences on perfusion quantification in Phase-Resolved Functional Lung (PREFUL) MRI.

**Materials and methods:**

Four cohorts of different subjects were imaged to analyze influences on the quantified perfusion maps: 1) at baseline and after 2 weeks to obtain the reproducibility (26 COPD patients), 2) before and after the administration of gadobutrol (11 COPD, 2 PAH and 1 asthma), 3) at 1.5T and 3T (12 healthy, 4 CF), and 4) with different acquisition sequences spoiled gradient echo (SPGR) and balanced steady-state free precession (bSSFP) (11 COPD, 7 healthy). Wilcoxon-signed rank test, Bland-Altman plots, voxelwise Pearson correlations, normalized histogram analyses with skewness and kurtosis and two-sample Kolmogorov-Smirnov tests were performed. *P* value ≤ 0.05 was considered statistically significant.

**Results:**

In all cohorts, linear correlations of the perfusion values were significant with correlation coefficients of at least 0.7 considering the entire lung (*P*<0.01). The reproducibility cohort revealed stable results with a similar distribution. In the gadolinium cohort, the quantified perfusion increased significantly (*P*<0.01), and no significant change was detected in the histogram analysis. In the field-strength cohort, no significant change of the quantified perfusion was shown, but a significant increase of skewness and kurtosis at 3T (*P* = 0.01). In the sequence cohort, the quantified perfusion decreased significantly in the bSSFP sequence (*P*<0.01) together with a significant decrease of skewness and kurtosis (*P* = 0.02). The field-strength and sequence cohorts had differing probability distribution in the two-sample Kolmogorov-Smirnov tests.

**Conclusion:**

We observed a high susceptibility of perfusion quantification to gadolinium, field-strength or MRI sequence leading to distortion and deviation of the perfusion values. Future multicenter studies should strictly adhere to the identical study protocols to generate comparable results.

## Introduction

Functional lung imaging has proven to offer valuable information for surveillance and early detection of various pulmonary diseases [1–4]. Techniques such as phase-resolved functional lung (PREFUL) MRI [5] avoid potential risks of contrast media, increase comfort and are already implemented clinically as they allow the calculation of ventilation- and perfusion-weighted maps enabling detection and monitoring various pulmonary diseases [6–9].

Recently, a perfusion quantification technique for PREFUL MRI was developed with good agreement to dynamic contrast-enhanced MRI in patients with chronic obstructive pulmonary disease (COPD) and chronic thromboembolic pulmonary hypertension in a single center setting [10]. However, a major challenge of medical imaging in general and especially of functional MRI, is the limited comparability across different sites, protocols and software. For instance, influences could be caused by the necessity of gadolinium contrast (GD) injection when these images serve as a primary endpoint of a study. Comparability might also be limited in multi-site studies with MR scanners using differing field-strengths. Furthermore, specific advantages of the imaging sequences could be exploited, for example higher SNR of a balanced steady-state free precession (bSSFP) sequence or fewer off-resonance artifacts of a spoiled gradient echo (SPGR) sequence at higher field-strengths. This would bring more flexibility across different sites and protocols. These influences on the signal are highly complex, which challenges absolute quantification of functional parameters as they are based on stable reference signals [11, 12].

Therefore, the aim of this study is the evaluation of the influences of previously applied gadolinium-based contrast media, a differing field-strength and a differing imaging sequence for the perfusion quantification of PREFUL MRI.

## Materials and methods

### Participants

**Fig 1:**
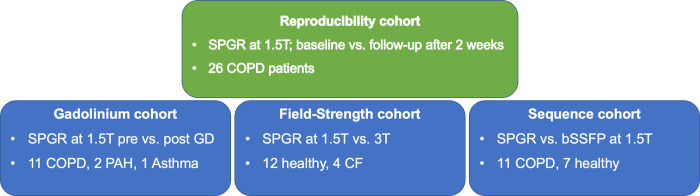
Overview of the used sequences and field-strengths across the four different cohorts. Spoiled gradient echo (SPGR), balanced steady-state free precession (bSSFP), chronic obstructive pulmonary disease (COPD), pulmonary arterial hypertension (PAH), cystic fibrosis (CF).

In this study, datasets of 74 participants from four cohorts were evaluated (Fig 1). All datasets were analyzed retrospectively. The study was approved by the local ethics committee ‘Ethikkommission der Medizinischen Hochschule Hannover’, and written informed consent was obtained from all study participants.

The reproducibility cohort was imaged at baseline and after two weeks. The cohort consisted of 26 patients with COPD (7 females, mean age 64 ± 7 years).

The gadolinium cohort was imaged before and after the administration of gadolinium-based contrast media and consisted of 11 patients with chronic obstructive pulmonary disease (COPD, 5 females, mean age 68 ± 6 years), 2 patients with pulmonal arterial hypertension (PAH, 1 female, mean age 65 ± 4 years) and one female patient with asthma (age 78 years).

The field-strength cohort was imaged at 1.5 Tesla (T) and 3T scanners and consisted of 12 healthy participants and 4 patients with cystic fibrosis (8 females, mean age 26 ± 7 years).

The sequence cohort was imaged with a SPGR and with a bSSFP sequence and consisted of 7 healthy participants and 11 patients with COPD (6 females, mean age 51 ± 18 years).

Inclusion criteria were as follows: COPD patients ≥ 40 years with stable cardiovascular function and a smoking history of ≥ 10 years were included. PAH patients ≥ 18 years with suspected pulmonary hypertension in transthoracic echocardiography were included. Patients with known cystic fibrosis were included. Healthy participants ≥ 18 years of age were included. Participants with contraindications to undergo MRI (e.g., due to pregnancy, claustrophobia or a pacemaker) or with lung infection or participation in other trials within the last 4 weeks were excluded.

### Imaging

The participants were scanned in head-first supine position to image a coronal slice at the level of the carina during free breathing. Parallel imaging with generalized autocalibrating partial parallel acquisition (GRAPPA) with acceleration factor 2 was performed. All images were interpolated to the final in-plane resolution of 256 × 256 pixels before reconstruction. The non-uniform intensity profiles of the applied receiver coils were corrected using the sensitivity profiles of the surface and body coil before further analysis. The optimal flip angle (Ernst angle) was used together with minimized time of repetition (TR) and echo time (TE) to maximize the MR-signal and to reduce scan time in each protocol. For further details please see following subsections.

#### Reproducibility cohort

The participants were imaged at 1.5T using a SPGR sequence (MAGNETOM Avanto n = 26, Siemens Healthineers, Erlangen, Germany). The follow-up measurement was acquired after two weeks using identical parameters: 50 cm x 50 cm field of view, 128 x 128 matrix size, 15 mm slice thickness, 0.82 ms TE, 3 ms TR, 5° flip angle and 1500 Hz/pixel bandwidth.

#### Gadolinium cohort

The participants were imaged at 1.5T using a SPGR sequence (MAGNETOM Aera n = 6 or MAGNETOM Avanto n = 8, Siemens Healthineers, Erlangen, Germany). The 1^st^ measurement was acquired before an MR-angiography with i.v. application of 0.1 mmol/kg gadobutrol. The 2^nd^ measurement started a mean 43 ± 1.9 s after the application. The SPGR sequence parameters were: 50 cm x 50 cm field of view, 128 x 128 matrix size, 15 mm slice thickness, 0.82 ms TE, 3 ms TR, 5° flip angle and 1500 Hz/pixel bandwidth.

#### Field-strength cohort

The participants were imaged at 1.5T and 3T scanners using a SPGR sequence (MAGNETOM Avanto and MAGNETOM Skyra, Siemens Healthcare, Erlangen, Germany) within 3 days. The examinations were performed at 1.5T with the following parameters: 50 cm x 50 cm field of view, 128 x 128 matrix size, 15 mm slice thickness, 0.82 ms TE, 3 ms TR, 5° flip angle and 1500 Hz/pixel bandwidth. Differing at 3T, the TE was 0.74, the TR was 1.83 and the flip angle was 4°.

#### Sequence cohort

The participants were imaged at 1.5T (MAGNETOM Avanto, Siemens Healthcare, Erlangen, Germany). A SPGR sequence was used for the 1^st^ and a bSSFP sequence was used for the 2^nd^ measurement. The SPGR sequence parameters were: 50 cm x 50 cm field of view, 128 x 128 matrix size, 15 mm slice thickness, 0.82 ms TE, 3 ms TR, 5° flip angle and 1500 Hz/pixel bandwidth. Differing for the bSSFP sequence, the TE was 0.64 ms, the TR was 1.41 ms, the flip angle was 35° and the bandwidth was 2055 Hz/pixel.

### Image registration

Linear and diffeomorphic image registration was performed by advanced normalization tools (ANTs) with a group-oriented registration scheme (GOREG) to achieve a uniform respiratory position for each image [13, 14]. The first 20 images of each sequence were included to analyze the signal decay towards the steady-state for quantification [10]. Furthermore, the corresponding series in each cohort were registered to allow for voxel-wise comparison between baseline and follow-up, pre and post gadolinium, 1.5T and 3T and SPGR and bSSFP sequences, respectively. Image-guided filtering was applied to all images of every dataset using an edge-preserving filter [15]. The images were segmented automatically using a convolutional neuronal network including the entire lung [16].

### Quantified perfusion mapping

As described previously [5], respiratory signal changes were excluded from the analysis by the application of a high pass filter at 0.75 Hz. The cardiac phases were estimated by piecewise fitting the averaged signal of a region of interest within the aorta to a cosine function. The images were then sorted by their phase and interpolated to an equidistant time grid. The signal difference between the time with the highest and the lowest signal of the time series in the parenchyma ROI was considered as the perfusion signal (Q) caused by flow-related enhancement [5].

Perfusion was quantified (Q_Quant_) by estimating the proton density and considering the median height of the signal decay towards the steady-state [10]. For this, the signals at several time points (t) of the time series were extracted to estimate the proportion of spins being exchanged per heart beat (exchange fraction) and the proportion of blood per voxel (blood fraction), which were combined together with the time interval between two heartbeats (T) and additional scaling factors (S1 Fig):

$$1)\;Q_{Quant}=exchange\;fraction\times blood\;fraction\times voxel\;volume\times \frac{1}{T}\times \frac{100}{2\times voxel\;volume}\;$$

$$2)\;Q_{Quant}=\frac{Q}{(S0par-S1par)\#}\times \frac{S0par}{S0ves\#}\times \frac{1}{T}\times \frac{100}{2}$$

The voxel volume is cancelled out. Q [a.u.] is the voxelwise signal change during the heart cycle. S0par [a.u.] is the voxelwise signal in a mid-respiratory parenchyma ROI extrapolated at time = 0. S1par # [a.u.] is the median signal in a mid-respiratory parenchyma ROI during the steady state. S0ves # [a.u.] is the median signal of vessel voxels extrapolated at time = 0 (99th percentile of voxels inside the lung ROI including vessels). T [s] is the time between two heart beats. Values indicated with # are determined globally for the whole imaging slice. To stay consistent with literature [17], the perfusion is quantified in ml/min/100 ml and therefore multiplied by 100. The factor of ½ accounts for the opposing flow directions of arterial and venous blood.

### Statistics

Statistical analysis was performed using MATLAB R2020b (The MathWorks, Natick, MA). Shapiro-Wilk test revealed no normal distribution of all functional parameters. Non-parametric paired Wilcoxon signed-rank test was used to assess significant differences of Q_Quant_, Q, S0par, S0ves, S1par and the heart frequency between the corresponding acquisitions. Differences between parameter medians with *P* < 0.05 were considered as significant. Bland-Altman plots were created and voxelwise Pearson correlation was calculated to compare the quantified perfusion (Q_Quant_) between both series in each cohort. The relative distribution of the quantified perfusion parameter was evaluated by histogram analyses. To ensure visual comparability, the perfusion values were normalized by dividing each value by the 95^th^ percentile of the values. Skewness and kurtosis were calculated for each histogram after excluding zeros and values over 1 to increase robustness against extreme outliers. Bin-range of the histograms was set to 0.01. Additionally, the value distributions of the normalized quantified perfusion of both corresponding series were evaluated using a two-sample Kolmogorov-Smirnov test.

## Results

**Fig 2:**
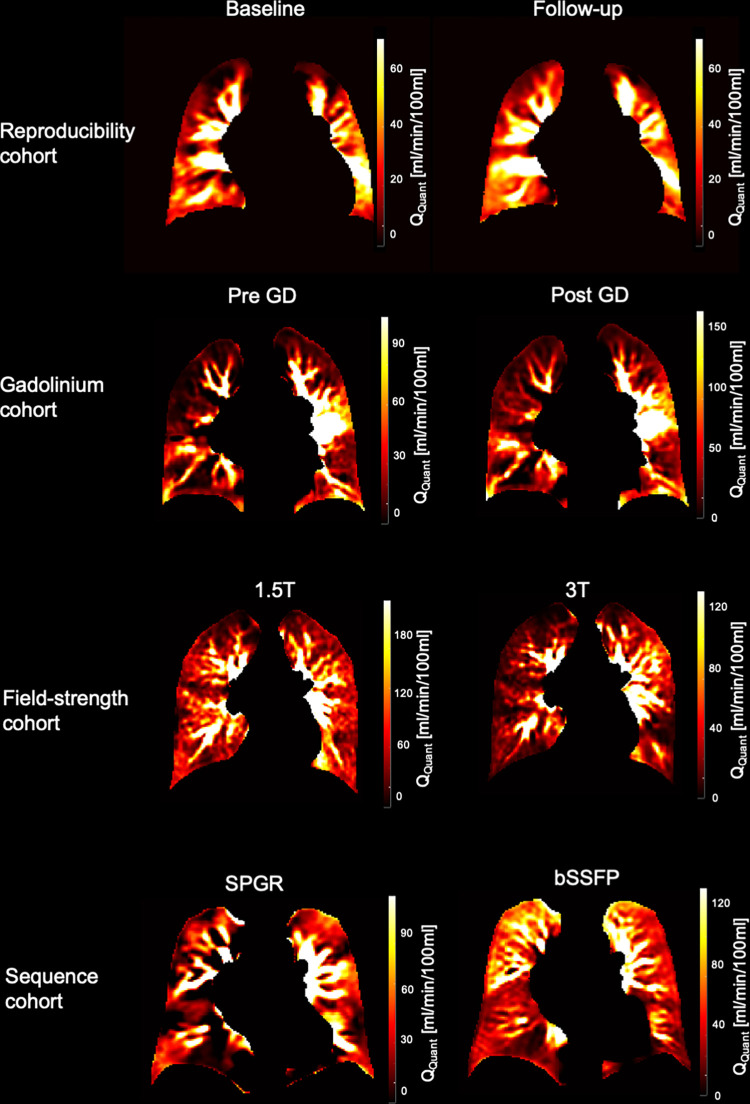
Representative quantified perfusion maps from each cohort. In the reproducibility cohort a 52-year-old female with COPD and minor changes after two weeks. In the gadolinium cohort a 64-year-old female with COPD and increased values after GD. In the field-strength cohort an 18-year-old female with CF lower values at 3T. In the sequence cohort a 50-year-old-female patient with COPD and fewer low values.

Fig 2 shows representative quantified perfusion maps of each cohort. First of all, the heart frequency presented no significant change between the corresponding series in any cohort (S2 Table).

### Reproducibility cohort

**Fig 3:**
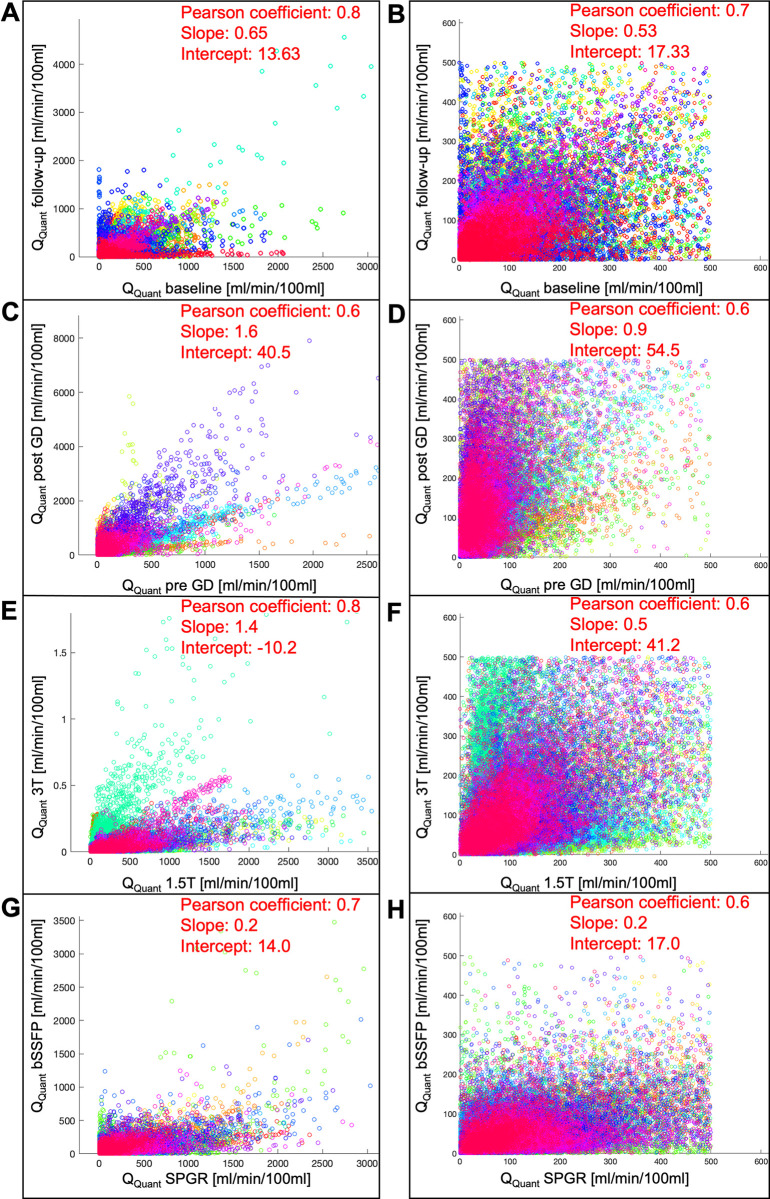
Voxelwise correlation of Q_Quant_ of each cohort. Patients are marked in different colors. Extreme values represent central lung vessels which were included in A, C, E, G. Values over 500 ml/min/100ml were excluded in B, D, F, H to evaluate only the distribution within the lung parenchyma. The reproducibility cohort is depicted in A&B, the gadolinium cohort in C&D, the field-strength cohort in E&F and the sequence cohort in G&H.

**Fig 4:**
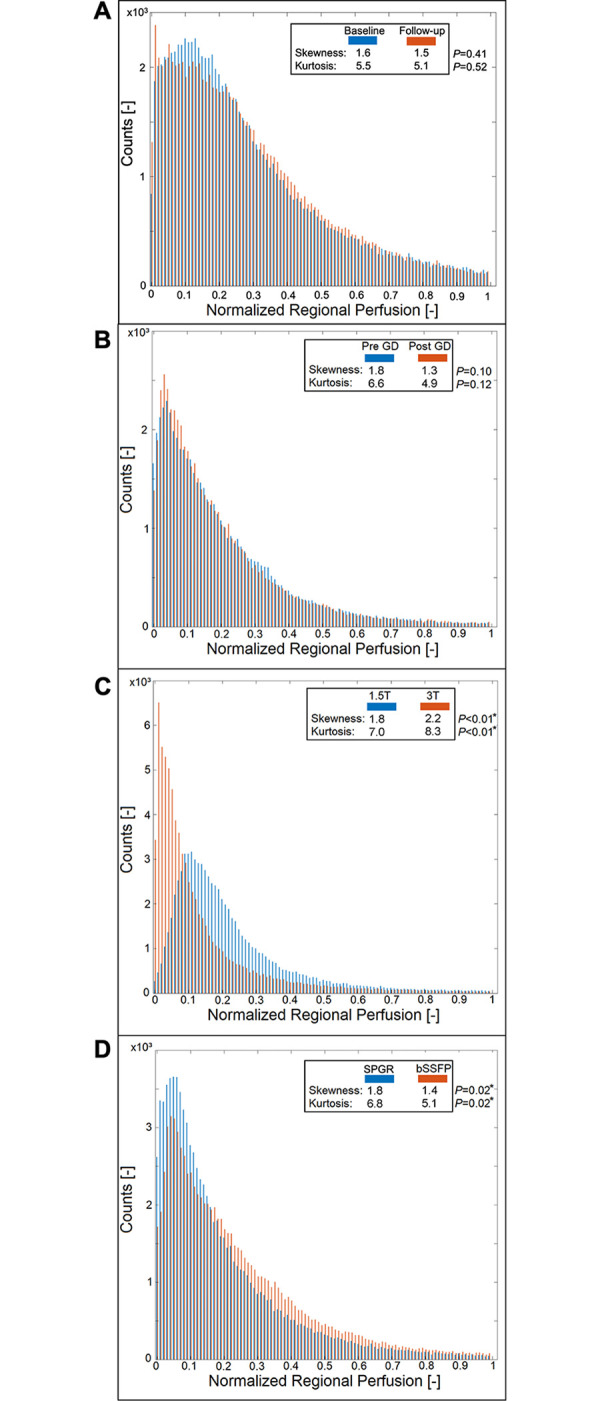
Histogram analyses of normalized Q_Quant_ of each cohort. Similar distribution of Q_Quant_ values at baseline and follow-up and before and after gadobutrol injection is seen in A and B. Major differences of the value distribution are visible between 1.5T and 3T with a downshift of the values at 3T in C. Smaller differences between the value distributions of both sequences with relatively fewer small values for the bSSFP are depicted in D.

No significant change of quantified perfusion was observed within the reproducibility cohort (*P* = 0.73) (Table 1). The Bland-Altman plot showed a mean difference of 0.21 ml/min/100ml with a standard deviation of 8.20 ml/min/100ml and a mean average of 19.69 ml/min/100ml between both series (S2 Fig). Voxelwise Pearson correlation of Q_Quant_ between both series was significant with a correlation coefficient of 0.8 (Fig 3 and Table 2). No change was detected for the value distribution in the normalized histogram analysis comparing skewness and kurtosis (*P* ≥ 0.41) or in the probability distribution using a two-sample Kolmogorov-Smirnov test (Fig 4 and S1 Table).

**Table 1:** Median values of the perfusion quantification ± interquartile range.

Parameter	Baseline	Follow-up	Wilcoxon-signed rank test
**Q**_**Quant**_ **[ml/min/100ml]**	**21.2 ± 13.6**	**21.6 ± 12.9**	***P* = 0.73**
	**Pre GD**	**Post GD**	
**Q**_**Quant**_ **[ml/min/100ml]**	**29.1 ± 13.7**	**67.5 ± 47.7**	*P* < 0.01*
	**1.5T**	**3T**	
**Q**_**Quant**_ **[ml/min/100ml]**	**79.7 ± 30.6**	**60.9 ± 70.3**	***P* = 0.20**
	**SPGR**	**bSSFP**	
**Q**_**Quant**_ **[ml/min/100ml]**	**35.9 ± 35.0**	**23.7 ± 18.0**	*P* < 0.01*

Significant *P*-values are marked with *.

**Table 2:** Median regional Pearson correlations of Q_Quant_ values ± interquartile range in each cohort.

Correlation of Q_Quant_	Baseline/Follow-up	Intercept	Significance
All values	0.8 ± 0.2	13.6	*P* < 0.01*
Values under 500 ml/min/100ml	0.7 ± 0.3	17.3	*P* < 0.01*
	**Pre GD/Post GD**	**Intercept**	
All values	0.7 ± 0.3	40.5	*P* < 0.01*
Values under 500 ml/min/100ml	0.6 ± 0.3	54.5	*P* < 0.01*
	**1.5T/3T**	**Intercept**	
All values	0.8 ± 0.1	-10.2	*P* < 0.01*
Values under 500 ml/min/100ml	0.6 ± 0.2	41.2	*P* < 0.01*
	**SPGR/bSSFP**	**Intercept**	
All values	0.7 ± 0.1	14.0	*P* < 0.01*
Values under 500 ml/min/100ml	0.6 ± 0.2	17.0	*P* < 0.01*

A threshold of 500 ml/min/100ml was chosen to exclude perfusion values of voxels in large central lung vessels. Values under this threshold were considered as voxels showing mostly parenchymal perfusion. Significant *P*-values are marked with *.

### Gadolinium cohort

The quantified perfusion increased significantly from 29.1 pre GD to 67.5 ml/min/100ml post GD (*P* < 0.01) (Table 1). While the perfusion amplitude Q and the S0-signals in the parenchyma and the vessel decreased significantly after the administration of gadolinium, the steady-state signal S1par increased significantly from 157.5 to 195.8 (S2 Table). The Bland-Altman plot showed a mean difference of -36.0 ml/min/100ml with a standard deviation of 34.9 ml/min/100ml and a mean average of 46.1 ml/min/100ml between both series (S2 Fig). Voxelwise Pearson correlation of Q_Quant_ between both series was significant with a correlation coefficient of 0.7 (Fig 3 and Table 2). No change was detected for the value distribution in the normalized histogram analysis with a decrease of the skewness from 1.8 to 1.3 (*P =* 0.10) and a decrease of the kurtosis from 6.6 to 4.9 (*P* = 0.12). Also, the two-sample Kolmogorov-Smirnov test revealed no differing probability distribution of the histogram values of both series (Fig 4 and S1 Table).

### Field-strength cohort

No significant change of the quantified perfusion was shown (*P =* 0.20) (Table 1). While the perfusion amplitude Q and the S0-signal in the parenchyma and the steady-state signal in the parenchyma decreased significantly at 3T, the S0-signal in the vessel increased significantly from 1496.7 to 2265.5 (S2 Table). The Bland-Altman plot showed a mean difference of 3.7 ml/min/100ml with a standard deviation of 85.7 ml/min/100ml and a mean average of 81.9 ml/min/100ml between both series (S2 Fig). Voxelwise Pearson correlation of Q_Quant_ was significant with a correlation coefficient of 0.8 (Fig 3 and Table 2). In the normalized histogram analysis, a pronounced difference between both series is visible with a downshift of the perfusion values at 3T (Fig 4). The skewness and kurtosis increased significantly from 1.8 to 2.2 and from 7.0 to 8.3. In accordance, the two-sample Kolmogorov-Smirnov test revealed a significantly differing probability distribution of the histogram values of both series (S1 Table).

### Sequence cohort

In the sequence cohort, the quantified perfusion decreased significantly from 35.9 using SPGR to 23.7 ml/min/100ml using bSSFP (*P* < 0.01) (Table 1). Also, all other parameters decreased significantly using the bSSFP (S2 Table). The Bland-Altman plot showed a mean difference of 24.3 ml/min/100ml with a standard deviation of 29.3 ml/min/100ml and a mean average of 38.2 ml/min/100ml between both series (S2 Fig). Voxelwise Pearson correlation of Q_Quant_ was significant with a correlation coefficient of 0.7 (Fig 3 and Table 2). In the normalized histogram analysis, a small difference between both series is visible with an upshift of the perfusion values in the bSSFP sequence (Fig 4). The skewness and kurtosis decreased significantly from 1.8 to 1.4 and from 6.8 to 5.1 and the two-sample Kolmogorov-Smirnov test revealed a significantly differing probability distribution of the histogram values of both series (S1 Table).

## Discussion

Our results indicated high regional correlation of the quantified perfusion parameters between all corresponding series. However, injection of gadobutrol, changing the field-strength or the sequence leads to significant alterations of the median values and/or the value distribution. In contrast, the reproducibility cohort with identical settings revealed quite stable median values together with a similar value distribution after two weeks.

The regional correlations of Q_Quant_ showed significant results supported by the vast quantity of the used data points. The different slopes and spread of extremely high values in Fig 3, which represent central vessels, might be the result of differing anatomy, central flow velocities and heart function between the patients or could display a systematic error of the method. After excluding values >500 ml/min/100ml, the correlation coefficients were lower (Fig 3). A reason for this could be a more accurate registration of regions with high contrasts such as vessels allowing better voxel-matching resulting in higher correlations. Nevertheless, all cohorts presented markedly deviations of the slope and high intercepts when correlating both corresponding series.

Only the reproducibility and gadolinium cohorts presented the same probability distribution in the two-sample Kolmogorov-Smirnov test and no significant change of the skewness and kurtosis. This is likely due to the use of the identical sequence parameters and field-strength. The application of gadolinium-based contrast agents and the following shortening of the T1-time led to a significant increase of the steady-state signal. Together with the signal decrease of the signals at the beginning of the sequence (t = 0), significantly increased quantified perfusion values were calculated. However, it is important to note, that the S0-values are not measured values, but extrapolated from the signal decay. Therefore, the decrease of the S0-values after GD could be the result of a lower extrapolation as a result of the increasing steady-state signal level (S1par). The same effect is present for S0ves, which is weaker, due to the velocity-dependence of the T1-time as described previously [10].

To achieve comparable values, while assuming a differing scan delay after injecting the contrast media, it would be necessary to use individual scaling factors as a function of time since injection and the individual elimination time, which is mostly dependent on the kidney function [18]. This factor would asymptotically decrease towards 1 during the elimination of the contrast over time. Otherwise, the T1-time would have to be considered in the perfusion model, which would necessitate T1-mapping for each patient. This approach could potentially also reduce variations due to differing T1 from varying hematocrit or oxygenation, although the latter is irrelevant at 1.5T [19].

The field-strength cohort showed no significant difference of the median height of the quantified perfusion parameter. Nevertheless, the interquartile range presented a markedly increase at 3T for all parameters, especially for the vessel signal at the beginning of the sequence (S0ves). Likely, the increasing spread of the values is caused by increasing field inhomogeneities occurring in lung MRI on interfaces such as vessel walls at increasing B0 [20]. The observed signal reduction within the parenchyma (S0par and S1par) at 3T could be the result of increasing susceptibility artifacts and spin-spin interactions shortening the T2*-relaxation time, while the signal in the vessel (S0ves) is increasing due to the gain of the SNR [20–23].

The perfusion amplitude (Q) decreases at the bSSFP sequence. However, the sequence parameters of the bSSFP could be further optimized to increase the overall lung signal and its variation over time [24, 25]. The high sensitivity of the bSSFP sequence to flow effects might lead to fewer zero-values within the perfusion maps causing lower skewness and kurtosis in the histogram analysis. Furthermore, the shorter TR and TE might improve sampling of the cardiac cycle enabling the detection of smaller signal differences. Taken together, the bSSFP sequence is very useful for functional imaging given its fast acquisition with sensitivity to flow effects, its high SNR and high contrast between fluids and other structures. However, its complex signal behavior with a mixed PD and $\sqrt{T2/T1}$ contrast during the transient phase and $\sqrt{T2/T1}$ contrast during steady-state causes a differing value distribution as the SPGR sequence [26].

A limitation of our study are the differing sequence parameters between 1.5T and 3T. A smaller flip angle of 4° was used at 3T to compensate for the increasing T1 of blood [27]. Nevertheless, changing field-strength, sequence parameters and hardware together makes an isolated evaluation of a single variable impossible.

Another limitation of our study is the variability of patient groups in each cohort, which could be affected differently. For instance, the COPD patients in the gadolinium cohort could be affected less due to their reduced blood volume and tissue density. There are however to few patient groups to make a subgroup analysis in this cohort. The field-strength cohort is composed of mostly healthy subjects and presents a relatively small deviation of the median values. This makes the difference in the value distribution even more remarkable since the large reproducibility cohort with 26 COPD patients showed a similar value distribution after two weeks. The same argument can be applied to the sequence cohort, which is composed of COPD patients and healthy participants, but also shows significant differences in median values and value distribution.

Taken together, the gadolinium, field-strength and sequence cohorts presented distortions of the quantified perfusion parameter, which may be challenging to correct. Perfusion quantification is influenced by various technical and physiological circumstances, such as inaccuracy of the estimated receive coil sensitivities, the angle of blood flow towards the imaging plane, image registration, field inhomogeneities or physiological perfusion variability [28]. Considering the similar value distribution, only the gadolinium cohort offers potential for a correction factor to compensate for the value increase, which however would necessitate additional measurements. The relatively stable results of the reproducibility cohort are noteworthy, as COPD patients tend to show more variability than healthy participants. This undermines the effects of gadolinium, field-strength and sequence on the perfusion quantification in the other cohorts. To date, PREFUL evaluations using a SPGR at 1.5T are validated best. Thus, the results show, that future multicenter studies should strictly adhere to an identical imaging protocol to generate comparable results across all sites. Also, parameters, that are derived from Q_Quant_ such as the perfusion defect percentage (QDP) would deviate due to the distortion of the value distributions [10].

In conclusion, the results indicate severe alterations and distortions of the quantified perfusion parameters after gadolinium administration and depending on the field-strength and on the applied sequence type.

## Supporting information

S1 Fig Theoretical model for the described perfusion quantification. The blood fraction is estimated by dividing the signal of the S0par voxel by the S0ves. The exchange fraction is estimated by dividing the amplitude of the perfusion signal Q by the median signal decay in the parenchyma ROI between t = 0 and the steady-state (DeltaS).
(TIF)

S2 Fig Bland-Altman plots of Q_Quant_ of each cohort. The average of Q_Quant_ of both corresponding series is on the x-axis and the difference is on the y-axis. The red solid line represents the mean bias and the dotted red lines the mean bias ± 1.96 standard deviation. In the reproducibility group (A), 26 patients with COPD in blue at baseline and after 2 weeks. In the gadolinium cohort (B), patients with PAH are marked in red, patients with COPD in blue and the patient with bronchial asthma in green. In the field-strength cohort (C), patients with cystic fibrosis are marked in green and the healthy participants in black. In the sequence cohort (D), patients with COPD are marked in blue and the healthy participants in black.
(TIF)

S1 Table Skewness and Kurtosis of the normalized Q_Quant_ values ± interquartile range in the histogram analysis. Significant *P*-values are marked with *. The distribution of the normalized Q_Quant_ values of both series were compared by a two-sample Kolmogorov-Smirnov test.
(DOCX)

S2 Table Median values of the variables for perfusion quantification ± interquartile range. Significant *P*-values are marked with *.
(DOCX)
