# Supplementary figures and images for: Influence of gadolinium, field-strength and sequence type on quantified perfusion values in phase-resolved functional lung MRI
Source: PLoS One. 2023 Aug 1;18(8):e0288744. doi: 10.1371/journal.pone.0288744 (PMC10393130; doi:10.1371/journal.pone.0288744)

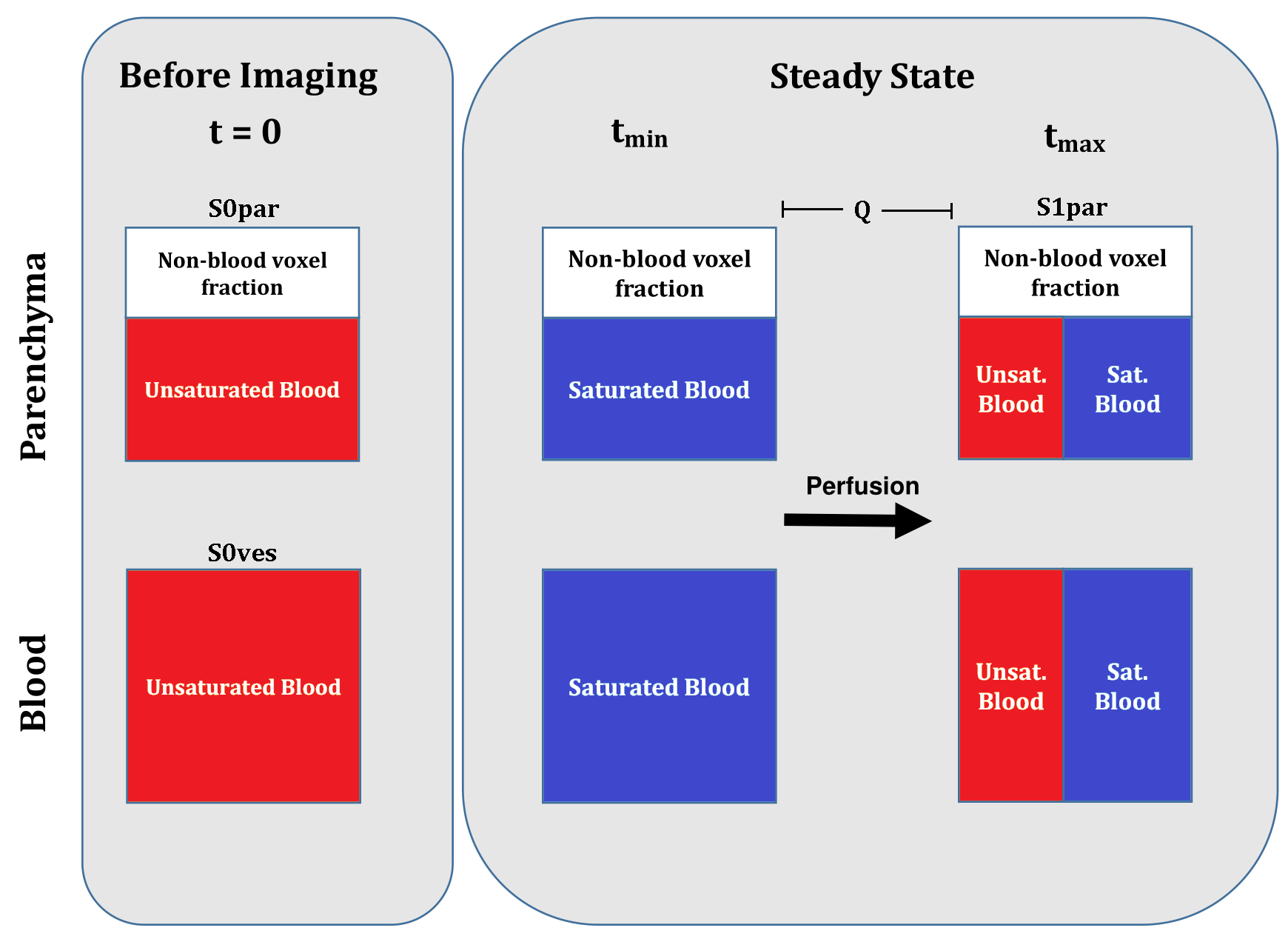

Supplement: S1 Fig — The blood fraction is estimated by dividing the signal of the S0par voxel by the S0ves. The exchange fraction is estimated by dividing the amplitude of the perfusion signal Q by the median signal decay in the parenchyma ROI between t = 0 and the steady-state (DeltaS). (TIF) [file pone.0288744.s001.tif]

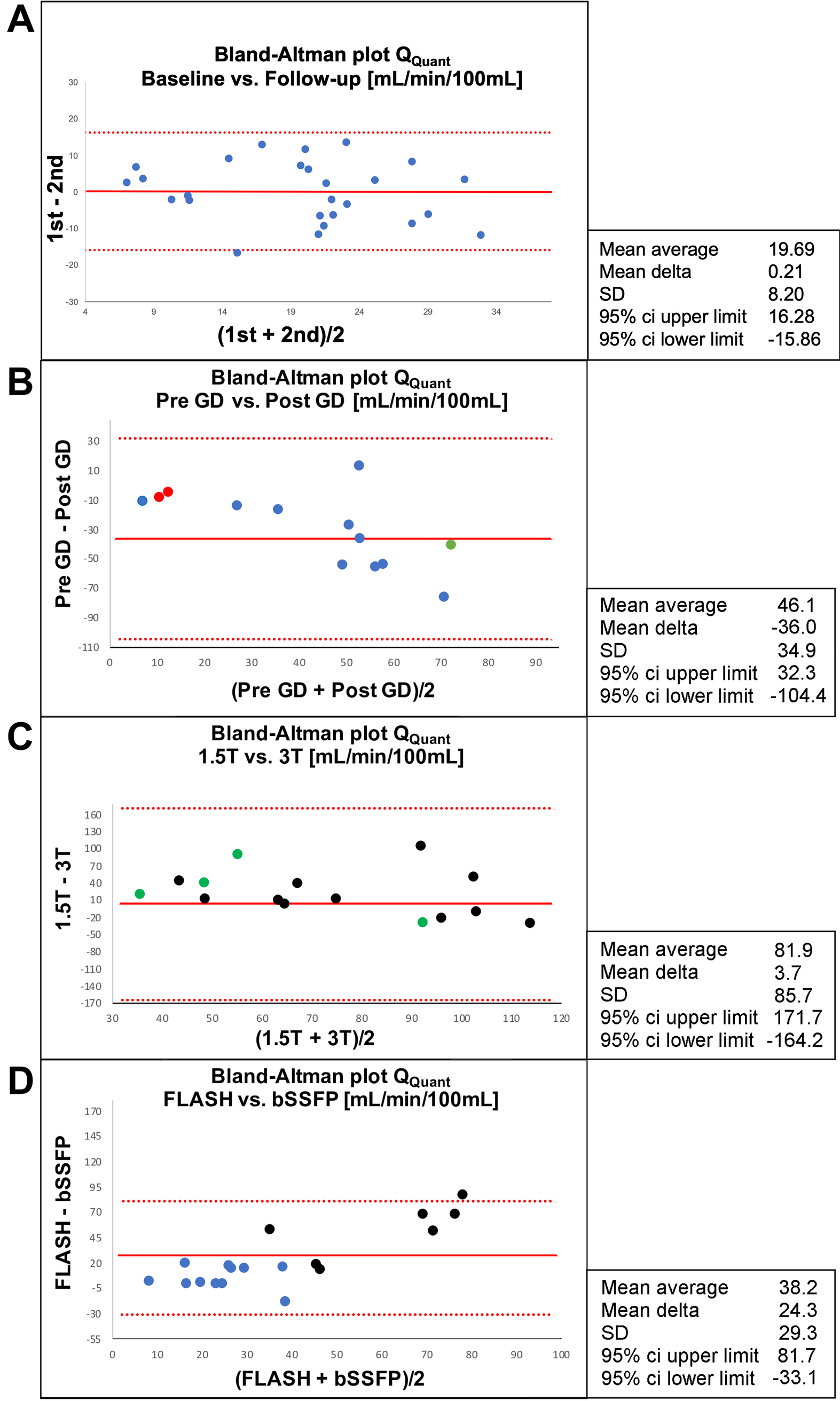

Supplement: S2 Fig — The average of QQuant of both corresponding series is on the x-axis and the difference is on the y-axis. The red solid line represents the mean bias and the dotted red lines the mean bias ± 1.96 standard deviation. In the reproducibility group (A), 26 patients with COPD in blue at baseline and after 2 weeks. In the gadolinium cohort (B), patients with PAH are marked in red, patients with COPD in blue and the patient with bronchial asthma in green. In the field-strength cohort (C), patients with cystic fibrosis are marked in green and the healthy participants in black. In the sequence cohort (D), patients with COPD are marked in blue and the healthy participants in black. (TIF) [file pone.0288744.s002.tif]
