# Supplementary material for: Influence of gadolinium, field-strength and sequence type on quantified perfusion values in phase-resolved functional lung MRI
Source: PLoS One. 2023 Aug 1;18(8):e0288744. doi: 10.1371/journal.pone.0288744 (PMC10393130; doi:10.1371/journal.pone.0288744)
Supplement: S1 Table — Significant P-values are marked with *. The distribution of the normalized QQuant values of both series were compared by a two-sample Kolmogorov-Smirnov test. (DOCX) [file pone.0288744.s003.docx]

| **Histogram of Q_Quant_** | **Baseline** | **Follow-up** | **Wilcoxon-signed rank test** | **Two-sample Kolmogorov-Smirnov test** |
| --- | --- | --- | --- | --- |
| Skewness | 1.6 ± 0.4 | 1.5 ± 0.7 | *P* = 0.41 | *Same probability distribution* |
| Kurtosis | 5.4 ± 2.3 | 5.1 ± 2.3 | *P* = 0.52 |  |
|  | **Pre GD** | **Post GD** |  |  |
| Skewness | 1.8 ± 0.8 | 1.3 ± 0.9 | *P =* 0.10 | *Same probability distribution* |
| Kurtosis | 6.6 ± 3.5 | 4.9 ± 3.0 | *P* = 0.12 |  |
|  | **1.5T** | **3T** |  |  |
| Skewness | 1.8 ± 0.6 | 2.2 ± 0.3 | *P* < 0.01* | *Differing probability distribution* |
| Kurtosis | 7.0 ± 2.2 | 8.3 ± 1.0 | *P* < 0.01* |  |
|  | **FLASH** | **bSSFP** |  |  |
| Skewness | 1.8 ± 0.4 | 1.3 ± 0.5 | *P* = 0.02* | *Differing probability distribution* |
| Kurtosis | 6.8 ± 1.5 | 5.1 ± 2.2 | *P* = 0.02* |  |

**S1 Table. Skewness and Kurtosis of the normalized Q_Quant_ values ± interquartile range in the histogram analysis.** Significant *P*-values are marked with *. The distribution of the normalized Q_Quant_ values of both series were compared by a two-sample Kolmogorov-Smirnov test.
