# Supplementary material for: Influence of gadolinium, field-strength and sequence type on quantified perfusion values in phase-resolved functional lung MRI
Source: PLoS One. 2023 Aug 1;18(8):e0288744. doi: 10.1371/journal.pone.0288744 (PMC10393130; doi:10.1371/journal.pone.0288744)
Supplement: S2 Table — Significant P-values are marked with *. (DOCX) [file pone.0288744.s004.docx]

| **Parameter** | **Pre GD** | **Post GD** | **Wilcoxon-signed rank test** |
| --- | --- | --- | --- |
| **Q_Quant_ [mL/min/100mL]** | **29.1 ± 13.7** | **67.5 ± 47.7** | ***P* < 0.01*** |
| Q [a.u.] | 4.9 ± 4.7 | 4.4 ± 3.4 | *P* = 0.03* |
| S0par [a.u.] | 290.2 ± 128.4 | 247.7 ± 98.6 | *P* < 0.01* |
| S0ves [a.u.] | 1378.8 ± 502.5 | 1334.0 ± 420.2 | *P* = 0.04* |
| S1par [a.u.] | 157.5 ± 55.1 | 195.8 ± 67.6 | *P* < 0.01* |
| Cardiac Frequency [1/s] | 1.2 ± 0.3 | 1.1 ± 0.4 | *P* = 0.98 |
|  | **1.5T** | **3T** |  |
| **Q_Quant_ [mL/min/100mL]** | **79.7 ± 30.6** | **60.9 ± 70.3** | ***P* = 0.20** |
| Q [a.u.] | 13.9 ± 4.4 | 12.2 ± 3.9 | *P* < 0.01* |
| S0par [a.u.] | 431.9 ± 98.9 | 246.1 ± 119.8 | *P* < 0.01* |
| S0ves [a.u.] | 1496.7 ± 336.6 | 2265.5 ± 2065.0 | *P* < 0.01* |
| S1par [a.u.] | 263.8 ± 40.3 | 182.4 ± 40.2 | *P* < 0.01* |
| Cardiac Frequency [1/s] | 1.1 ± 0.3 | 1.1 ± 0.2 | *P* = 0.79 |
|  | **SPGR** | **bSSFP** |  |
| **Q_Quant_ [mL/min/100mL]** | **35.9 ± 35.0** | **23.7 ± 18.0** | ***P* < 0.01*** |
| Q [a.u.] | 6.0 ± 2.8 | 3.1 ± 2.2 | *P* < 0.01* |
| S0par [a.u.] | 245.7 ± 55.8 | 156.7 ± 66.9 | *P* < 0.01* |
| S0ves [a.u.] | 1077.7 ± 313.3 | 657.1 ± 245.3 | *P* = 0.04* |
| S1par [a.u.] | 156.7 ± 37.9 | 71.8 ± 18.2 | P < 0.01* |
| Cardiac Frequency [1/s] | 1.1 ± 0.1 | 1.1 ± 0.2 | P = 0.87 |

**S2 Table. Median values of the variables for perfusion quantification ± interquartile range.** Significant *P*-values are marked with *.
